# Impact of antibody framework residue V_H_-71 on the stability of a humanised anti-MUC1 scFv and derived immunoenzyme

**DOI:** 10.1038/sj.bjc.6601759

**Published:** 2004-04-06

**Authors:** J Krauss, M A E Arndt, Z Zhu, D L Newton, B K Vu, V Choudhry, R Darbha, X Ji, N S Courtenay-Luck, M P Deonarain, J Richards, S M Rybak

**Affiliations:** 1 SAIC, National Cancer Institute at Frederick, Frederick, MD 21702, USA; 2 Laboratory of Experimental and Computational Biology, National Cancer Institute at Frederick, Frederick, MD 21702, USA; 3 Macromolecular Crystallography Laboratory, National Cancer Institute at Frederick, Frederick, MD 21702, USA; 4 Antisoma Research Ltd, West Africa House, Hanger Lane, Ealing W5 3QR, UK; 5 Imperial College of Science, Technology & Medicine, London SW7 2AZ, UK; 6 Developmental Therapeutics Program, National Cancer Institute at Frederick, Frederick, MD 21702, USA

**Keywords:** scFv, stability, ribonuclease, MUC1, HMFGl, fusion protein

## Abstract

Anti-MUC1 single-chain Fv (scFv) fragments generated from the humanised antibody huHMFG1 had adequate antigen-binding properties but very poor stability irrespective of the applied linker or domain orientation. Mutagenesis of heavy-chain framework residue V_H_-71, previously described as a key residue for maintaining the CDR-H2 main-chain conformation and thus important for antigen binding, markedly stabilised the scFv while having only a minor effect on the binding affinity of the molecule. Because of its improved stability, the engineered fragment exhibited immunoreactivity with tumour cells even after 7 days of incubation in human serum at 37°C. It also showed, in contrast to the wild-type scFv, a concentration-dependent binding to the target antigen when displayed on phage. When fusing the scFv to the recombinant ribonuclease *rap*LRI, only the fusion protein generated with the stable mutant scFv was able to kill MUC1^+^ tumour cells with an IC_50_ of 80 nM. We expect this novel immunoenzyme to become a promising tool for the treatment of MUC1^+^ malignancies.

The MUC1 membrane mucin glycoprotein is overexpressed in most adenocarcinomas (Taylor-Papadimitriou *et al*, 1999) and associated with poor prognosis in patients with many epithelial cancers, including colorectal and gastric carcinoma (Utsunomiya *et al*, 1998; Baldus *et al*, 2002). Recently, MUC1 was also found to be overexpressed in a variety of haematological malignancies including acute myelogenous leukaemia, chronic lymphocytic leukaemia, and multiple myeloma (Brossart *et al*, 2001). Since glycosylation of MUC1 in cancer cells is distinct from mucin expressed in healthy tissue (Hanisch and Muller, 2000), tumour-associated mucin represents a valuable target for diagnostic and therapeutic approaches with monoclonal antibodies (mAbs). Several mAbs have been raised against the highly conserved immunogenic core region of the extracellular domain of MUC1, which comprises tandem repeats of 20 amino acids (Gendler *et al*, 1988). These include mAb HMFG1 (Taylor-Papadimitriou *et al*, 1981), which recognises the PDTR epitope of the protein core with high specificity (Price *et al*, 1990). HMFG1 becomes internalised after antigen binding (Aboud-Pirak *et al*, 1988) and thus provides a valuable tool for the selective delivery of cytotoxic agents into tumour cells. A ^90^Y-HMFG1 radioimmunoconjugate was employed in a phase I–II clinical trial in patients with ovarian cancer in an adjuvant setting. Intraperitoneal administration of a single dose of the reagent resulted in a >10 years long-term survival of 78% of these patients (Epenetos *et al*, 2000). A multicentre, multinational, phase III clinical trial utilising ^90^Y-HMFG1 for the adjuvant treatment of women with ovarian cancer completed enrolment in 2003, and first results are expected during 2004. To overcome the immunogenicity of mAb HMFG1 and make the antibody suitable for repeated systemic administration, a humanised version, designated huHMFG1, was generated by grafting the murine antigen-binding site into human antibody frameworks (Verhoeyen *et al*, 1993). huHMFG1 was shown to retain the antigen affinity and same specificity of the rodent ancestor. Results from a recently completed radioimmunoimaging study in breast cancer patients (Al-Yasi *et al*, 2002) showed the specific binding of huHMFG1 to tumour tissues. The humanised antibody is currently being evaluated as a therapeutic agent in a phase I clinical trial in patients with metastatic breast cancer. For many clinical applications, such as radioimmunoimaging, radioimmunotherapy, or administration of recombinant cytotoxic fusion proteins, the employment of small antigen-binding fragments such as single-chain Fv (scFv) antibodies or multivalent derivatives may have advantages over the use of antibodies in the IgG format. ScFv fragments penetrate solid tumour tissue more efficiently (Yokota *et al*, 1992) and are rapidly cleared from the circulation (Milenic *et al*, 1991). To exploit these advantages, huHMFG1 was reformatted into an scFv fragment. Although this construct exhibited appropriate antigen binding, its half-life was <2 h when incubated in human serum at 37°C. Low biophysical stability of scFv fragments was shown to be associated with the failure to localise to xenografted tumour tissue in immunodeficient mice (Willuda *et al*, 1999). Thus, for clinical applications, sufficient stability of scFv fragments is of paramount importance.

The aim of this study was to generate a humanised anti-MUC1 scFv with sufficient antigen-binding and stability properties as required for clinical applications. Here we show that mutagenesis of human antibody framework 3 residue V_H_-71Arg to the corresponding murine donor antibody site alanine (V_H_-71Arg → Ala) dramatically increased the stability of the humanised scFv while only having a minor impact on the affinity of the molecule. As a consequence of its improved stability, only the robust mutant scFv genetically fused to the ribonuclease *rap*LRI mediated cytotoxicity towards tumour cells and recognised its native target antigen when displayed on phage. Our results indicate that framework residue V_H_-71 plays a critically important role in providing intrinsic stability to antibody heavy-chain variable domains.

## MATERIALS AND METHODS

### Identification of unusual framework residues

To identify unusual residues within the human variable domain framework regions (FRs), which could possibly influence the structural integrity of the grafted antigen-binding site of the humanised scFv antibody, we aligned its amino-acid sequence to sequence reference templates. Mismatching ‘key residues’ (Chothia *et al*, 1989) between the frameworks of the murine donor antibody sequence and the human acceptor antibody sequence were identified and canonical-class assignments of the donor antibody complementarity determining regions (CDRs), L1–L3, H1, and H2, respectively, were determined by screening the sequence against sequence templates of antibody repertoires (Martin and Thornton, 1996) at http://www.bioinf.org.uk/. Furthermore, uncommon residues at the V_H_/V_L_ interface (Chothia *et al*, 1985) of the humanised scFv with a potential to compromise interdomain stability of the molecule were inspected.

### Generation of wild-type scFv huHMFG1 and scFv mutants

To generate a humanised scFv, the variable light chain and the variable heavy chain of huHMFG1 were PCR amplified from plasmids pAS1 and pAS2 (Dr R Young, Antisoma Research Ltd), containing the complete light chain and heavy chain of the humanised antibody, respectively. Single-chain Fv variants with flanking *Nco*I and *BamH*I restriction sites were generated by PCR in either domain orientation and with different linker peptides (Table 1

**Table 1:** huHMFG1 scFv variants

**Clone**				
**V_H_71R**	**V_H_71A**	**Orientation**	**Linker length**	**Linker sequence**
17W^a^	17M^a^	V_L_–V_H_	17	ASSGGGGSGGGGSGGSA
22W	22M	V_L_–V_H_	22	ASSGGGGSGGGGSGGSAGGGGS
4.10W	4.9M	V_H_–V_L_	15	GGGGSGGGGSGGGGS

a W=wild type; M=mutant.

). Mutations were introduced by oligonucleotide directed mutagenesis and overlap extension PCR techniques as described elsewhere (Ho *et al*, 1989). Fragments were cloned into vector pHOG21 (Kipriyanov *et al*, 1996) for the expression of soluble protein.

### Periplasmic expression and purification of scFv fragments

Single-chain Fv fragments were expressed as soluble protein in the periplasm of the *Escherichia coli* strain TG1 (Stratagene, La Jolla, CA, USA) and purified by immobilised metal chelate affinity chromatography (IMAC) and size-exclusion chromatography as described previously (Arndt *et al*, 2003). Concentrations of purified scFvs were determined spectrophotometrically from the absorbance at *A*_280 nm_ using the extinction coefficient ε^1mg ml^−1^^ =1.67.

### Generation, expression, and purification of scFv-ribonuclease fusion proteins

**Figure 1:**
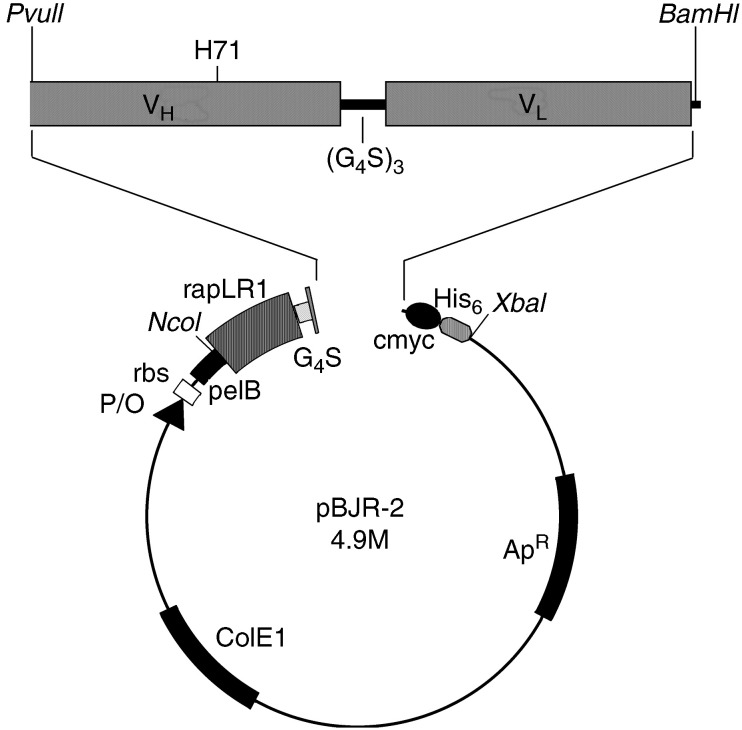
Schematic representation of fusion protein expression vector pBJR-2. Ap^R^, ampicillin resistance gene; ColE1, origin of DNA replication; c-myc, sequence encoding the c-myc epitope; His_6_, hexa-histidine encoding sequence; P/O, *lac* wild-type promoter/operator; rbs, ribosome-binding site; pelB, signal peptide sequence of bacterial pectate lyase; V_H_, variable heavy chain; V_L_, variable light chain; rapLR1, *Rana pipiens* liver ribonuclease 1; H71, framework 3 residue 71; G_4_S, spacer connecting the ribonuclease with the scFv; (G_4_S)_3_, linker connecting the variable domains. Restriction sites for cloning are indicated.

Expression vector pBJR-2 was generated by cloning the PCR-amplified gene encoding the amphibian ribonuclease *rap*LRI (Chen *et al*, 2000) and G_4_S spacer with flanking *Nco*I and *Pvu*II restriction sites into the expression vector pHOG21 (Kipriyanov *et al*, 1996). *Rap*LR1-G_4_S-4.9M was obtained by ligating the *Pvu*II*–Bam*HI fragment of scFv 4.9M into the *Pvu*II*–Bam*HI-restricted pBJR-2 vector (Figure 1

). The correct size and sequence of the insert were analysed by restriction digest and DNA sequencing, respectively.

*E. coli* TG1 bacteria transformed with the plasmid encoding the *rap*LR1-G_4_S-4.9M gene were grown at 37°C overnight in 50 ml 2YT media containing 200 *μ*g ml^−1^ ampicillin and 2% glucose. Bacteria were diluted into 4 l 2YT media containing 200 *μ*g ml^−1^ ampicillin and grown at 37°C to an OD_600_ of 1.0. Protein expression was induced by adding IPTG to a final concentration of 1.0 mM to the culture and incubation continued at 26°C for 16–18 h. The bacteria were centrifuged at 10 000 **g** for 45 min at 4°C. The fusion protein was isolated from inclusion bodies, denatured, renatured, and dialysed as described previously (Newton *et al*, 1996). The extensively dialysed protein solution was applied to a 40 ml CM-Sephadex C-50 column (Pharmacia Biotech Inc., Piscataway, NJ, USA) equilibrated with 20 mM Tris-HCl, pH 7.5, containing 10% glycerol. The fusion protein was eluted with equilibration buffer containing 1.0 M NaCl. The protein solution was diluted 10-fold with 50 mM NaOAc, pH 5.0, immediately applied to a 2 ml SP-Sepharose column (Pharmacia Biotech Inc.) and eluted with a 200 ml linear NaCl gradient (0.1–0.7 M) in 50 mM NaOAc, pH 5.0.

### Cell-binding assays

Specific binding of scFv constructs was determined by flow cytometry using the human MUC1^+^ cell lines MCF7 (ATTC, Manassas, VA, USA) and SKOV-3 (ATTC), and mouse myeloma B-cell line Sp2/0-Ag14 (ATTC) and human T-cell line Jurkat (ATTC) as a negative control. For the determination of antigen-binding affinity constants (*K*_d_), MCF7 cells were used. Staining of antibody fragments and affinity measurements were performed as described previously (Benedict *et al*, 1997). Stained cells were analysed on a FACScan Flow Cytometer (BD Bioscience, San Jose, CA, USA), and median fluorescence intensity (MFI) was calculated using the CellQuest™ software (BD Bioscience). Background flourescence was subtracted. Equilibrium constants were determined by using the Marquardt–Levenberg algorithm for nonlinear regression with the GraphPad Prism version 3.0a for Macintosh (GraphPad Software, San Diego, CA, USA).

### Cytotoxicity assay

Cytotoxicity of scFv-RNase fusion proteins towards target cells was analysed by a protein synthesis inhibition assay. In all, 2.5 × 10^3^ MCF7 cells in a volume of 100 *μ*l were plated in each well of a 96-well plate 24 h before treatment and the assay performed according to standard procedures as described previously (Newton *et al*, 1996). Experiments were performed at least twice. The concentration of fusion protein test sample that inhibits protein synthesis by 50% (=IC_50_) was determined from semilogarithmic plots as percentage of [^14^C]leucine incorporation into mock-treated cells.

### Generation of computer homology models

Three homologous models of scFv 4.9M were generated based on existing structures of scFv proteins (Berman *et al*, 2000) with either an Arg or an Ala at position V_H_-71 located in the heavy-chain FR 3. Two forms of huHMFG1-Arg71 with distinct conformations in their CDR-H1 (residues 31–35) and CDR-H2 (residues 50–65) regions were modelled based on observed conformations in scFv structures with an Arg at position V_H_-71. In one form, the side chain of V_H_-71Arg is exposed to the surface of the protein as observed in PDB entry 1NQB (Pei *et al*, 1997), whereas in the other, this side chain is buried between motifs CDR-H1 and CDR-H2 as in PDB entry 2FBJ (Suh *et al*, 1986). Only one form of scFv huHMFG1-Ala71 was modelled because all the scFv structures with an Ala at V_H_-71 exhibit the same conformation as in PDB entry 1EO8 (Fleury *et al*, 2000).

Model-1 with exposed V_H_-71Arg side chain was built based on a trimeric antibody (PDB 1NQB; Pei *et al*, 1997) showing 63% sequence similarity to scFv huHMFG1. The starting model was constructed with 1NQB fragments C121–C233 of the primary molecule and C1–C120 of a symmetry mate. Mutations were performed according to the sequence of scFv huHMFG1. Model-2 with the buried V_H_-71Arg side chain was built based on the crystal structure of antibody J539 (PDB entry 2FBJ; Suh *et al*, 1986). The sequence similarity of J539 with scFv huHMFG1 is very low (54%). Hence, fragments FR3 (V_H_-66-94) and CDR-H3 (V_H_-95-102) and the V_L_ region were taken from Model-1 followed by the introduction of corresponding mutations. Model-3 with V_H_-71Ala was built based on PDB entry 1EO8 (Fleury *et al*, 2000). The V_H_ and V_L_ sequences of 1EO8 are 72 and 26% identical with those of huHMFG1, respectively. Mutations were performed in the V_H_ region; the V_L_ region was taken from Model-1. Modelling was performed using the graphics program O (Jones and Kjeldgaard, 1997), the models were optimised using the energy minimisation procedure embedded in the program CNS (Brünger and Rice, 1997), and the illustration was made using programs Bobscript (Esnouf, 1997) and Raster3D (Merritt and Bacon, 1997).

### Expression of scFv fragments on phage

Wild-type sequence scFv 4.10W and mutant scFv 4.9M encoding genes (Table 1) or wild-type scFv 17W with randomised linker peptide sequence were cloned into phagemid pCANTAB-5 (Amersham Pharmacia, Uppsala, Sweden) after the introduction of flanking restriction sites *Sfi*I/*Not*I by PCR. Phagemid virions were prepared essentially according to standard methods as described (Sambrook *et al*, 1989; Marks *et al*, 1991).

### Phage ELISA

The ability of phage-displayed scFvs for recognising the target antigen MUC1 was determined by whole-cell ELISA with MCF7 cells. In all, 1 × 10^4^ cells well^−1^ were seeded in a 96-well plate (Nunc Nalgene, Roskilde, Denmark) and grown in DMEM medium (Invitrogen, Carlsbad, CA, USA) supplemented with 10% fetal bovine serum until 60–70% confluent. Cells were fixed by adding paraformaldehyde diluted in PBS to a final concentration of 1% and incubated for 10 min at room temperature. After washing with PBS, a serial dilution of purified scFv phagemid virion or M13KO7 helper phage alone (Invitrogen) was added to the cells. Plates were gently shaken for 2 h at 37°C. After 6 × washing with PBS/Tween-20 0.02%, rabbit anti-M13-HRP polyclonal antibody (1 : 5000; Amersham Pharmacia Biotech Inc., Piscataway, NJ, USA) was added. The mixture was incubated at 37°C for 1 h. Cells were washed with PBS and bound phage scFvs were detected using ABTS (Sigma Chemicals, Saint Louis, MO, USA) as a substrate. After 30 min incubation, absorbance was read with an ELISA reader (Bio-Tek Instruments Inc., Winooski, VT, USA) at 405 nm.

### Biophysical stability

Single-chain Fv fragments were incubated at 37°C in 90% human serum at a concentration of 20 *μ*g ml^−1^ for up to 7 days. Samples were taken at different time points and stored at –20°C. Binding activity of the samples to MUC1^+^ MCF7 cells was determined by flow cytometry. The MFI was determined as described above. Temperature-dependent degradation of monomeric scFvs was determined by incubation of samples at 37°C in 90% PBS at a concentration of 20 *μ*g ml^−1^ for 1 h, followed by analytical gel filtration on a calibrated Superdex 75 HR10/30 column (Amersham Pharmacia Biotech Inc.)

## RESULTS

### Generation and characterisation of wild-type sequence scFvs

In order to generate a small antigen-binding molecule to be used as a building block for the subsequent construction of recombinant fusion proteins, the variable domains of light and heavy chains of the humanised mAb huHMFG1 (Verhoeyen *et al*, 1993) were recloned into a V_L_–V_H_ oriented scFv format, separated by a synthetic 17-amino-acid linker peptide (clone 17W, Table 1). Although the scFv showed specific binding to MUC1^+^ tumour cells, its half-life in human serum at 37°C was less than 2 h (data not shown). To improve the stability of the wild-type scFv molecule by alteration of the linker peptide, this region was randomised by PCR and the resulting scFv fragments were recloned into a phagemid vector for display on phage. However, no clones could be enriched after panning the generated scFv library on MUC1^+^ MCF7 cells. These results indicate that the linker-randomised scFvs were not displayed in a conformation for binding to native antigen. To further investigate whether the V_L_–V_H_ connecting linker peptide length or domain orientation accounted for the poor stability of the wild-type scFv, two further linker variants, 22W and 4.10W, were generated (Table 1). Both constructs retained specific binding to MUC1^+^ tumour cells but did not show an increase in their half-lives, indicating that changing the domain orientation or linker length was not sufficient to increase the stability of the molecules.

### Analysis of huHMFG1 variable domain wild-type sequence

The amino-acid sequence of the humanised scFv antibody was analysed as described in Materials and Methods. We identified V_H_-71, previously described as a ‘key residue’ for the CDR-H2 main-chain conformation (Chothia *et al*, 1989; Tramontano *et al*, 1990), to be potentially critical for maintaining the structural integrity of the grafted antigen-binding site of the wild-type scFv fragment. To study the effects of framework residue V_H_-71 on antigen-binding and stability properties of the humanised scFvs, three mutant variants were generated by replacing arginine in the human framework 3 sequence with the murine donor antibody residue alanine (clones 17M, 22M, 4.9M; Table 1).

### Effects of V_H_-71Arg → Ala mutation on stability and specificity of scFv fragments

**Figure 2:**
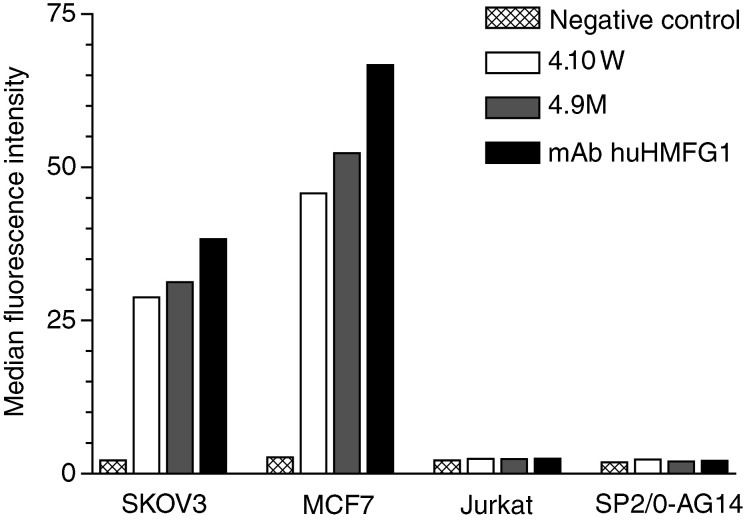
Specific binding activity of mAb huHMFG1 and derived scFv fragments 4.10W and 4.9M. Flow cytometric analysis revealed for all purified antibodies binding to breast carcinoma cell line MCF7 and ovarian carcinoma cell line SKOV3, no binding was detected for T-cell lymphoma cell line Jurkat and mouse myeloma B-cell line SP2/0-AG14. Cells stained with secondary antibodies only were used as negative control.

**Figure 3:**
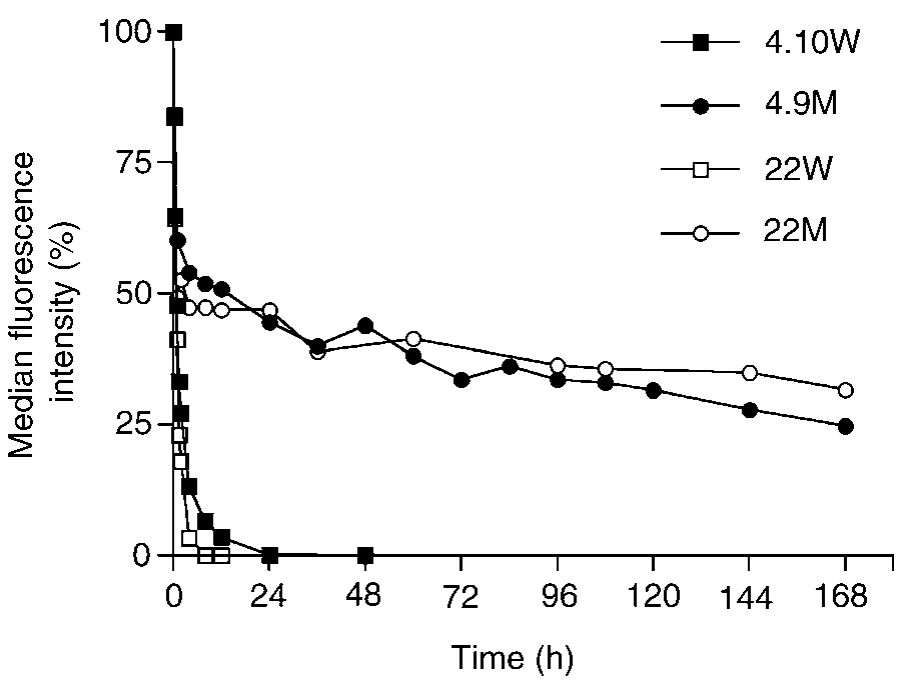
Serum stability of scFv fragments. Immunoreactivity of wild-type scFvs 4.10W and 22W, and mutant variant scFvs 4.9M and 22M with MCF7 cells was measured by flow cytometry after incubating the constructs at 37°C in human serum for various time periods. Binding activity is shown as per cent of the maximal MFI at time point zero.

**Figure 4:**
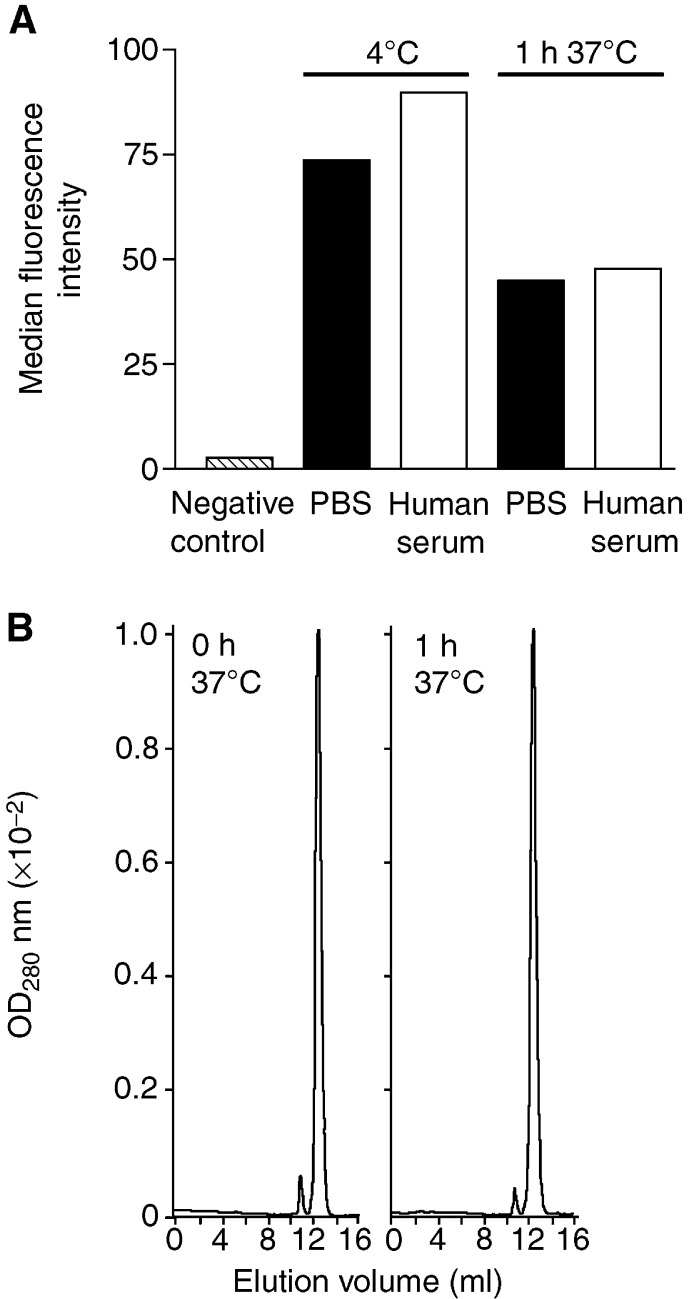
Effect of temperature on immunoreactivity and stability of mutant scFv 4.9M. (**A**) Binding was analysed by flow cytometry before and after incubation at 37°C for 1 h either in PBS or human serum. MCF7 cells stained with secondary antibodies were used as negative control. (**B**) Analytical size-exclusion FPLC on a calibrated Superdex 75 column was performed before (left panel) and after (right panel) incubation of 20 *μ*g ml^−1^ scFv in PBS at 37°C for 1 h. Monomeric protein eluted at 12.5 ml at a flow rate of 0.3 ml min^−1^.

For initial characterisation, the mutant variant scFvs (clones 17M, 22M, 4.9M; Table 1) were IMAC purified. In flow cytometry, all three variants showed specific binding to MUC1^+^ MCF7 cells. To assess the global stability, the engineered scFvs were incubated in human serum at 37°C for 12 h. The half-live of mutant 17M was not increased when compared with the corresponding wild-type scFv 17W (data not shown). In contrast, immunoreactivity with tumour cells at this time point was observed for mutants 22M and 4.9M, respectively. Monomers of scFvs 22M and 4.9M as well as their wild-type counterparts (clones 22W and 4.10W, respectively) were subsequently separated from higher molecular weight species by size-exclusion chromatography. Both purified wild-type and mutant scFvs exhibited specific binding towards MUC1^+^ tumour cells as shown in Figure 2

for the example of constructs in the V_H_-V_L_ orientation. The stability of 22M and 4.9M monomers was determined by incubation of purified proteins in human serum at 37°C for various time periods. As demonstrated in Figure 3

, both mutant scFvs exhibited dramatically improved stability when compared with the corresponding wild-type constructs. Nevertheless, both constructs showed a substantial decline in cell-binding activity within the first hour of serum incubation (4.9M, 40%; 22M, 51%) although they retained specific binding to tumour cells even after 7 days of serum incubation (Figure 3). To determine whether this phenomenon was induced by exposure of the scFvs to elevated temperature or to serum components, mutant scFv 4.9M was incubated in PBS for 1 h at 37°C and binding activity to MCF7 cells was analysed by flow cytometry. As shown in Figure 4A

, the median flourescence intensity dropped to a similar level whether the scFv was incubated in human serum or PBS at 37°C. Analytical size-exclusion chromatography revealed no degradation of the monomeric protein at this time point (Figure 4B), suggesting that the initial decrease in immunoreactivity was due to reduced binding affinity caused by exposure of the scFv to the elevated temperature.

### Effects of V_H_-71Arg → Ala mutation on affinity of scFv fragments

Binding affinity constants for the wild-type scFvs 22W and 4.10W, and mutant variants 22M and 4.9M (Table 1) were determined. As shown in Table 2

**Table 2:** Binding affinity constants of mAb, wild-type, and mutant scFv fragments

		**scFv**			
	**mAb huHMFG1**	**22W**	**4.10W**	**22 M**	**4.9 M**
*K*_d_ (nM)^a^	2.3	199	113	160	103
*K*_d_ (nM)^b^					195

a Affinity determined at room temperature.

b Affinity determined after 1 h incubation in PBS at 37°C.

, mutagenesis of V_H_-71Arg → Ala had only a small effect on binding affinity of the constructs to the target cells irrespective of the variable domain orientation and linker. Since a temperature-dependent decrease in immunoreactivity of the mutant scFv fragments with rapid onset was observed in the stability assay, the binding constant of the construct with the tightest antigen binding (clone 4.9M) was determined again after preincubation in PBS for 1 h at 37°C. This short exposure to physiological body temperature resulted in a 1.9-fold decrease in antigen-binding affinity (Table 2).

### Computer homology modelling

**Figure 5:**
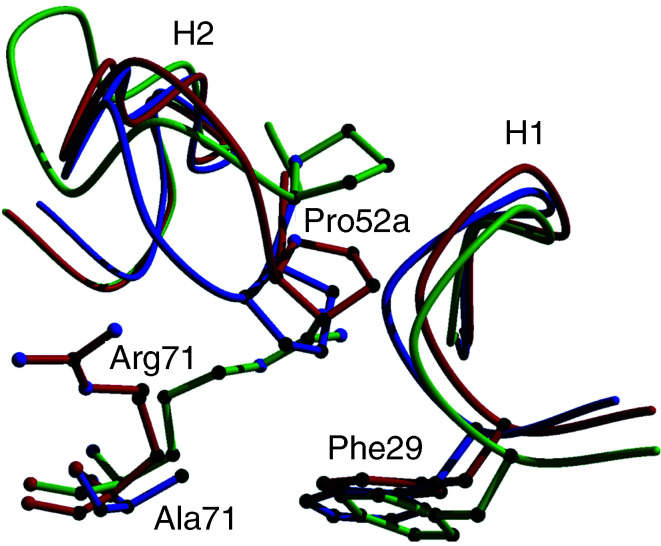
Homology modelling. The models shown as worm diagrams illustrate the influence of residue V_H_-71 on the conformation of CDR-H1 and CDR-H2. Model-1 with the side chain of Arg71 exposed to the surface of the V_H_ domain is shown in red, Model-2 with Arg71 buried between motifs CDR-H1 and CDR-H2 in green, and Model-3 with Ala71 in blue.

In order to understand the structural consequences of the V_H_-71Arg → Ala mutation, we analysed the impact of this mutation on the conformation and relative position of antigen-binding loops (CDR-H1 and CDR-H2) and calculated the buried surface area of the interface between the two motifs CDR-H1 and CDR-H2 for the three huHMFG1 models. As shown in Figure 5

, the backbone conformation of CDR-H1 is virtually the same in all three models, whereas the conformation of CDR-H2 is significantly different. However, it appears that the buried surface area between motifs CDR-H1 and CDR-H2 is dictated by the identity of residue V_H_-71 but not the conformation of the two motifs: For the V_H_-Ala71 model, it is 481 Å^2^, which is significantly larger than for the two V_H_-Arg71 models (384 and 398 Å^2^ in Model-1 and Model-2, respectively).

### Expression of scFv variants on phage

**Figure 6:**
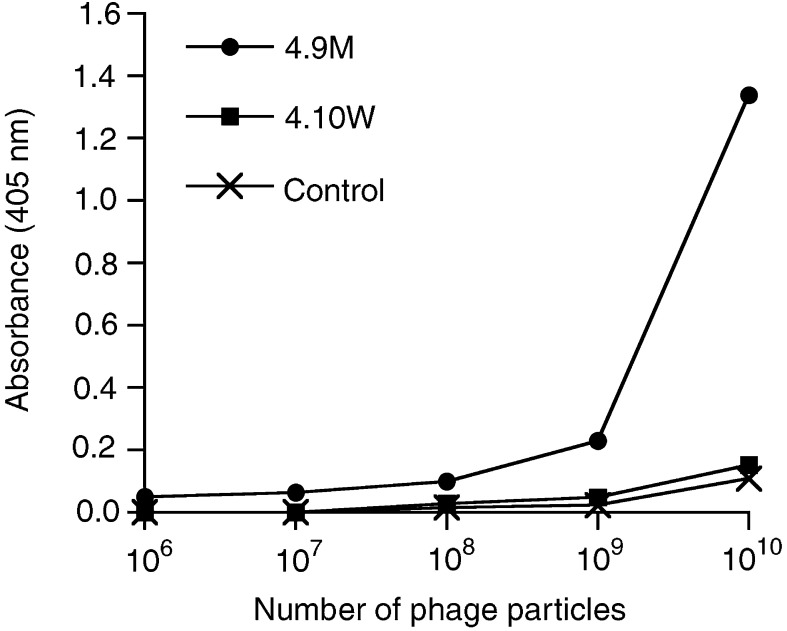
Whole-cell ELISA of phage-displayed wild-type and mutant scFv. Various concentrations of 4.10 wild-type scFv phage, 4.9 mutant scFv phage, or control M13KO7 helper phage were incubated with MCF7 cells subconfluently grown in 96-well plates for 2 h at 37°C. Bound phages were detected with anti-M13-HRP polyclonal antibody as described in Materials and Methods.

To examine the possibility of using the humanised scFv antibody as a template to select entire human fragments from phage display libraries by chain shuffling (Marks *et al*, 1992), both wild-type sequence scFv 4.10W and mutant 4.9M were recloned in a phagemid vector for display on phage. The titre of the concentrated phage stock was 1 × 10^12^−1 × 10^13^ CFU ml^−1^. In whole-cell ELISA, only the displayed mutant scFv 4.9M showed reactivity with MUC1^+^ tumour cells in a concentration-dependent manner (Figure 6

), indicating that the wild-type scFv was not appreciably expressed on phage.

### Effect of V_H_-71Arg → Ala mutation on cytotoxic properties of a recombinant immunoenzyme

**Figure 7:**
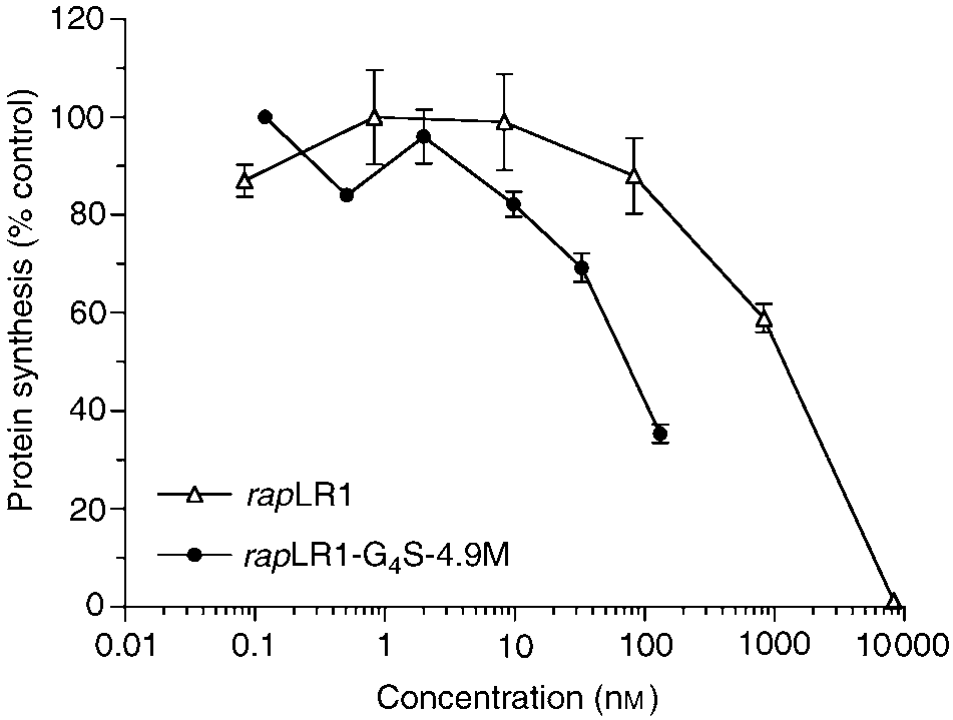
Cytotoxicity of *rap*LR1-G_4_S-4.9M fusion protein. Various concentrations of the fusion protein or RNase alone were incubated with MCF7 cells for 3 days and protein synthesis measured by [^14^C]leucine incorporation as described in Materials and Methods. Protein synthesis inhibition of target cells is expressed as percentage of protein synthesis to buffer-treated control cells.

To evaluate the stabilised humanised scFv antibody as a targeting moiety for the selective delivery of cytotoxic agents into tumour cells, a recombinant fusion protein comprising the recombinant ribonuclease *rap*LR1 and scFv 4.9M was made. This construct could be purified to homogeneity and was active in killing MCF7 cells with an IC_50_ of 80 nM (Figure 7

). The cytotoxic activity of *rap*LR1 in the fusion protein configuration was increased approximately 18-fold (IC_50_ for native *rap*LR1, 1500 nM). In contrast, a fusion protein comprising *rap*LRI and wild-type scFv 17W could not be purified to homogeneity and did not exhibit any cytotoxic activity towards MUC1^+^ tumour cells.

## DISCUSSION

The aim of this study was to generate a functional scFv molecule from the clinically established humanised anti-MUC1 mAb huHMFG1. For clinical applications, scFv molecules must have sufficient affinity for the target antigen and remain stable in the human body for at least several hours. Failure to meet these criteria was shown to result in insufficient enrichment of scFv molecules in xenografted solid tumours in immunodeficient mice (Adams *et al*, 1998; Willuda *et al*, 1999), thus hampering future clinical applications.

We initially generated an scFv molecule from the variable domains of the humanised mAb huHMFG1 that specifically bound to MUC1^+^ tumour cells but did not exhibit sufficient stability. It has been reported that the length and amino-acid composition of scFv linker peptides can influence both the stability and solubility of scFv fragments (Robinson and Sauer, 1998). To assess whether the linker peptide or domain orientation may have contributed to the poor stability of the wild-type sequence scFvs, we systematically varied linker length, domain orientation, and eventually amino-acid composition of the linker by a phage display approach. None of these approaches, however, resulted in the generation of a stable molecule.

Sequence analysis of the humanised wild-type scFv revealed framework 3 residue V_H_-71Arg to be potentially critical for disrupting the structural integrity of the grafted murine antigen-binding site. Site V_H_-71 is located at one of the two corners of the inner faces of the *β*-sheets and can be exposed to the solvent through the twist and coil of the *β*-sheet (Chothia *et al*, 1998). It is one of a set of sites forming the ‘Vernier’ zone (Foote and Winter, 1992). The ‘Vernier’ zone comprises a layer of framework residues that support antigen-binding loop conformations and their relative dispositions and has therefore been suggested to play an important role in fine-tuning the fit of an antibody to antigen (Foote and Winter, 1992). Residues with small side chains at position V_H_-71 such as an alanine provide a cavity for orienting the CDR-H2 loop in close proximity to CDR-H1 (Tramontano *et al*, 1990; Xiang *et al*, 1995). Disruption of the cavity by replacing a small side chain with bulkier ones was shown to separate the antigen-binding loops CDR-H1 and CDR-H2 from each other and expose the CDR-H2 loop residue V_H_52a to the surface (Tramontano *et al*, 1990). This resulted in a 12-fold decrease in the binding affinity of a chimeric antibody with specificity for the TAG72 antigen (Xiang *et al*, 1995), a >5-fold lower affinity of a humanised anti-ErB2 antibody for binding to breast cancer cells (Carter *et al*, 1992), and a complete loss of antigen binding of a humanised anti-alpha 4 integrin antibody (Leger *et al*, 1997). In contrast, crystal structures of several mutants of a humanised anti-lysozyme Fv fragment with different side-chain sizes of V_H_-71 revealed no conformational adjustment of CDR-H2 relative to CDR-H1 in either the ligand-free form or in complex with lysozyme (Holmes *et al*, 2001). In addition, V_H_-71 was reported to be of minor importance for antigen binding of a humanised anti-CD18 antibody (Hsiao *et al*, 1994). In this study, we also observed minor effects on antigen-binding properties of the V_H_-71Arg → Ala mutant scFv. Taken together, these data indicate that the residue type at V_H_-71 alone may not invariantly fix the spatial orientation of CDR-H1/CDR-H2 and thus may not always play an important role in influencing antigen-binding properties of an antibody. Surprisingly however, the mutant scFv fragments exhibited a 1.9-fold decline in antigen-binding affinity after short exposure to physiological body temperature. This phenomenon could be observed after preincubation at 37°C in either human serum or PBS. Because protein degradation could be excluded by analytical size-exclusion chromatography, it is conceivable that this phenomenon most likely occurred through a temperature-dependent conformational alteration of the antigen-binding site of the molecule.

The stabilised monovalent scFv antibody 4.9M exhibited 45-fold reduced binding affinity when compared with the parental humanised IgG. As shown for bivalent diabodies derived from scFvs with different affinities for binding to ErB2, the increment in binding affinity upon dimerisation was greatest for the lowest affinity scFv (65-fold) and least for the highest affinity scFv (7.7-fold) (Nielsen *et al*, 2000). In keeping with this observation, a phage display library-selected Fab fragment with low affinity for binding to MUC1 exhibited an even 160-fold improved apparent *K*_d_ after being re-engineered into a bivalent fully human IgG molecule (Henderikx *et al*, 2002). Similarly, reformatting the mutant scFv 4.9M characterised in this study into a bivalent diabody resulted in a molecule with tight antigen binding (*K*_d_ 8 nM; J Krauss, unpublished results). These data indicate that the moderate binding affinity of the scFv and derived fusion proteins can be markedly enhanced by generating bivalent derivatives.

Although residue V_H_-71 has been described as a major determinant for the CDR-H1 and CDR-H2 conformation and thus important for antigen binding, we are not aware of any reports indicating that this site also has an impact on the stability of antibody scFv fragments. As our data clearly show, the replacement of the bulky side-chain residue arginine by alanine at V_H_-71 dramatically increased the stability of the scFv fragments irrespective of the domain orientation. As a result, both mutant variants exhibited immunoreactivity with tumour cells even after 7 days of incubation in human serum at 37°C. The magnitude of stability improvement was surprising since the above-described location of V_H_-71 and its structural role did not necessarily suggest this residue to be of such critical importance for providing stability to the humanised scFv fragment. To study the consequences of the V_H_-71Arg → Ala mutation on the scFv structure, three computer homology models were generated. The models revealed the buried surface area between CDR-H1 and CDR-H2 to be significantly larger (approximately 20%) in the V_H_-71Ala variant, resulting in a markedly stabilised V_H_ domain of the mutant scFvs.

Since previous attempts to display the wild-type scFv huHMFG1 on phage were unsuccessful, we analysed whether this failure was caused by the insufficient stability of the fragment. Indeed, our data show that only the stable mutant scFv recognised native antigen, indicating that appropriate intrinsic stability of the molecule was absolutely required for being displayed on phage in correctly folded conformation. The possibility to express the mutant scFv on phage paves the way for generating entire human anti-MUC1 fragments by chain shuffling of a human phage display library.

Ribonucleases have become established potent anticancer reagents within the last few years. In a recently conducted multicentre phase II clinical trial, a ribonuclease very closely related to *rap*LRI used in this study, Ranpirnase (onconase), was administered to more than 100 patients with unresectable malignant mesothelioma (Mikulski *et al*, 2002). Overall response rates of about 50% were achieved. When given intravenously on a weekly basis, in some cases for more than 3 years, onconase was remarkably well tolerated in the vast majority of patients, with no appreciable adverse side effects attributed to immunogenicity of the reagent. Factors accounting for the low immunogenic potential of onconase may include its considerable sequence homology to human ribonucleases and the small size, basicity, and lack of glycosylation of the molecule. *Rap*LRI, as a very close homologue of onconase, might therefore not be expected to exhibit significant immunogenicity in humans despite its amphibian origin. We have previously shown that *rap*LRI exhibits cytotoxic properties comparable to those of onconase and that its potency could be dramatically increased both *in vitro* and *in vivo* after conjugation to mAbs (Hursey *et al*, 2002). In contrast to onconase, *rap*LRI does not require an N-terminal pyroglutamate in order to retain its full activity (Hursey *et al*, 2002), suggesting this ribonuclease to represent a most promising candidate for fusion with small-sized antibody fragments. By showing that only a recombinant *rap*LRI antibody fusion protein made with a stable scFv could be purified to homogeneity and exhibited potent cytotoxic activity, this study clearly illustrates the importance of addressing the stability properties of scFv fragments intended to be used for the generation of recombinant fusion proteins.

Since most recent results indicate strong *in vivo* activity of an immunoconjugate comprising the parental humanised mAb huHMFG1 and *rap*LRI in tumour-xenotransplanted immunodeficient mice (DL Newton, unpublished results), we believe that the novel, stable immunoenzyme derivative merits further investigation as a therapeutic agent for patients with MUC1^+^ malignancies.
